# Supplementary material for: Comparative genomics provides new insights into the diversity, physiology, and sexuality of the only industrially exploited tremellomycete: Phaffia rhodozyma
Source: BMC Genomics. 2016 Nov 9;17:901. doi: 10.1186/s12864-016-3244-7 (PMC5103461; doi:10.1186/s12864-016-3244-7)
Supplement: Additional file 6: — List of orphan genes with links to PFAM (related to Additional file 1: Table S1). (ZIP 1428 kb) [file 12864_2016_3244_MOESM6_ESM.zip › PFAM_UNIQUE/G04250_P.html]

#                                                                            --- full sequence --- -------------- this domain -------------   hmm coord   ali coord   env coord
  
# target name        accession   tlen query name           accession   qlen   E-value  score  bias   #  of  c-Evalue  i-Evalue  score  bias  from    to  from    to  from    to  acc description of target
  
#------------------- ---------- ----- -------------------- ---------- ----- --------- ------ ----- --- --- --------- --------- ------ ----- ----- ----- ----- ----- ----- ----- ---- ---------------------
  
G04250\_P             -            390 End3                 PF12761.2    195      0.58    8.4   4.0   1   2   0.00028      0.28    9.5   0.3    10    77    56   123    55   177 0.82 - Actin cytoskeleton-regulatory complex protein END3  
G04250\_P             -            390 End3                 PF12761.2    195      0.58    8.4   4.0   2   2      0.25   2.5e+02   -0.2   0.4    71   103   242   274   211   329 0.70 - Actin cytoskeleton-regulatory complex protein END3
